# Clinical and microbiological characteristics of pyogenic liver abscess in a tertiary hospital in East China

**DOI:** 10.1097/MD.0000000000008050

**Published:** 2017-09-15

**Authors:** Haishen Kong, Fei Yu, Weili Zhang, Xuefen Li

**Affiliations:** a State Key Laboratory for Diagnosis and Treatment of Infectious Diseases, Collaborative Innovation Center for Diagnosis and Treatment of Infectious Diseases; b Key Laboratory of Clinical In Vitro Diagnostic Techniques of Zhejiang Province, Department of Laboratory Medicine, First Affiliated Hospital, College of Medicine, Zhejiang University, Hangzhou, China.

**Keywords:** antibiotic resistance, *Klebsiella pneumonia*, pyogenic liver abscess

## Abstract

Pyogenic liver abscess (PLA) is a potentially life-threatening disease affecting many parts of the world, especially Asia. In this study, we explored the clinical and microbiological characteristics of PLA in Chinese patients.

A 5-year (2010–2014) retrospective review of medical records on all PLA patients who were admitted to a tertiary teaching hospital was performed.

Among 217 PLA cases who were confirmed cultural positive, *Klebsiella pneumonia* (*K pneumonia)* was the most common pathogen (n = 165, 76.0%), followed by *Escherichia coli* (n = 21, 9.7%). Notably, there is a higher incidence of diabetes mellitus in patients with *K pneumoniae*-induced PLA (KP-PLA) than that with non-*K pneumoniae*-induced PLA (non-KP-PLA)(43.0% vs 21.2%, *P* = .005). However, it was less prevalent for concomitant hepatobiliary disease (20.0% vs 34.6%, *P* = .039) and history of intraabdominal trauma or surgery (13.3% vs 38.5%, *P* < .001) in patients with KP-PLA. Although *K pneumoniae* are sensitive to most common antibiotics (antibiotic resistance rates below 10%), some strains (1.2%) developed resistant to carbapenem. These results confirmed *K pneumoniae* as the predominant pathogen of PLA in the area in which the study was conducted. More attention should be directed toward monitoring the emergence of carbapenem-resistant *K pneumoniae*.

KP-PLA is frequently diagnosed in patients with metabolic diseases accompanied by serious consequences, and it is therefore prudent to see that they receive sensitivity-directed antibiotic therapy.

## 1 Introduction

Pyogenic liver abscess (PLA) is a potentially life-threatening disease throughout the world, with incidences ranging from 1.1 to 17.6/100,000 individuals.^[1–4]^ A large population-based retrospective study in northeast China has reported the incidence rate of 5.7 per 100,000 population.^[5]^ The different pathogenic spectra of PLA may vary across different countries and areas. Knowing the etiology of PLA, when possible, plays an important role in the successful therapy of PLA patients. Recently, the prevalence of *Klebsiella pneumonia*-induced PLA (KP-PLA) has become an emerging public health problem all around the world.^[2,6–8]^ Although KP-PLA mainly appears in Asians, it is rarely reported that KP-PLA emerges in mainland China. The purpose of this study was to investigate the clinical and pathogenic features of PLA in East China through a 5 years retrospective review of medical records in a tertiary teaching hospital to learn the clinical features and microbiological characteristics of PLA more comprehensively, as well as provide basis and assistance for the prevention and treatment of PLA.

## 2 Materials and methods

### 2.1 Study design

A retrospective study based on a tertiary teaching hospital during a 5-year period was conducted from January 2010 to December 2014 to evaluate the clinical and microbiological characteristics of PLA.

### 2.2 Study patients and inclusion criteria

The medical and microbiological records of all of the patients who were hospitalized due to PLA and were treated at the first affiliated hospital of the medical college of Zhejiang University, a tertiary teaching hospital in East China were retrospectively reviewed. The patients with PLA included in our study met the following criteria: older than 18 years; the presence of the typical clinical symptoms of liver abscess including fever, chills, and liver pain; imaging evidence, including ultrasonography (US), computerized tomography (CT), and magnetic resonance imaging (MRI), that was consistent with a PLA; laboratory examinations, including white blood count, abnormal biomarkers and blood or pus culture; surgical findings. We excluded patients whose medical treatment data were incomplete or missing.

### 2.3 Data source

All of the parameters included in the investigation were collected in series by reviewing the patient medical records preserved in the Electronic Medical Record system. The patient records included demographic characteristics (age and sex), clinical parameters (signs and symptoms), laboratory values (hematologic, biochemical, and microbiological findings), radiological findings (solitary or multiple abscesses and lobar distribution), concomitant diseases, diagnoses, treatment procedures, catheter drainage, and outcomes at discharge (recovered or died). The microbiological parameters included in the present study consisted of polymicrobial infection, monomicrobial infection, anaerobic infection, and extended-spectrum β-lactamases (ESBLs) producing isolates. Polymicrobial infection was defined as the presence of ≥2 pathogens cultured from blood or pus specimens. The patient was considered to have anaerobic infection when anaerobic isolates were cultured from blood or pus.

### 2.4 Microbiology laboratory procedures

All microbiology samples, including blood and pus, were processed for bacterial culture in a central laboratory. The VITEK 2 Compact (bioMérieux, Marcy l’Etoile, France) were used to identify the bacterial isolates and 19 commonly used clinical antibiotics were analyzed using the K-B method. The antibiotics tested included amikacin, ampicillin, ampicillin/sulbactam, sulfamethoxazole/trimethoprim, ciprofloxacin, meropenem, gentamicin, tigecycline, ceftazidime, cefepime, cefazolin, cefuroxime, cefoxitin, ceftriaxone, aztreonam, imipenem, ertapenem, levofloxacin, and piperacillin/tazobactam. The ESBLs phenotype was confirmed for all the collected isolates by phenotypic confirmatory disc diffusion test according to the manual issued by the Clinical and Laboratory Standards Institute.

### 2.5 Statistical analysis

Statistical analysis was performed using the SPSS version 16.0 statistical software package. The descriptive data are here presented as the means with standard deviations (SDs) for continuous data and as percentages for categorical data. The *χ*^2^ test and Fisher exact test were used to evaluate the differences in the categorical variables. The Student *t* test was used to evaluate the differences in the continuous variables. *P* < .05 was considered statistically significant in all analyses. The drug resistance rate of main pathogen was analyzed statistically using WHONET 5.6 software.

## 3 Results

### 3.1 Clinical features of the study subjects

During the year of 2010 to 2014, getting access to the hospital discharge database, 339 patients were identified, among which 40 patients were excluded owing to lack of results on blood or pus culture. Ultimately, 299 patients were enrolled in this retrospective study in total. Demographic characteristics and clinical features of PLA patients are shown in Table 1. Males were predominant (n = 180, 60.2%), with a mean age of 55.9 ± 11.8 years. The majority of PLA patients had fevers and laboratory indexes indicating higher than normal C-reactive protein levels. Most cases of PLA were localized to the right hepatic lobe and affected ≥2 lobes. Eighty-one patients (27.1%) had a medication history of hepatobiliary disease, 72 patients (24.1%) had intra-abdominal trauma or surgery before, and 34 patients (11.3%) had been diagnosed as malignancy previously. As for the method of abscess drainage, surgical drainage was used in 24 patients and percutaneous drainage was used in 275 patients. Although empirical antibiotics and abscess drainage intervention were carried out in all PLA patients, 38 patients (12.7%) developed septic shock, 60 patients (20.1%) turned into intensive care unit (ICU), and 4 patients (1.3%) died finally.

**Table 1:**
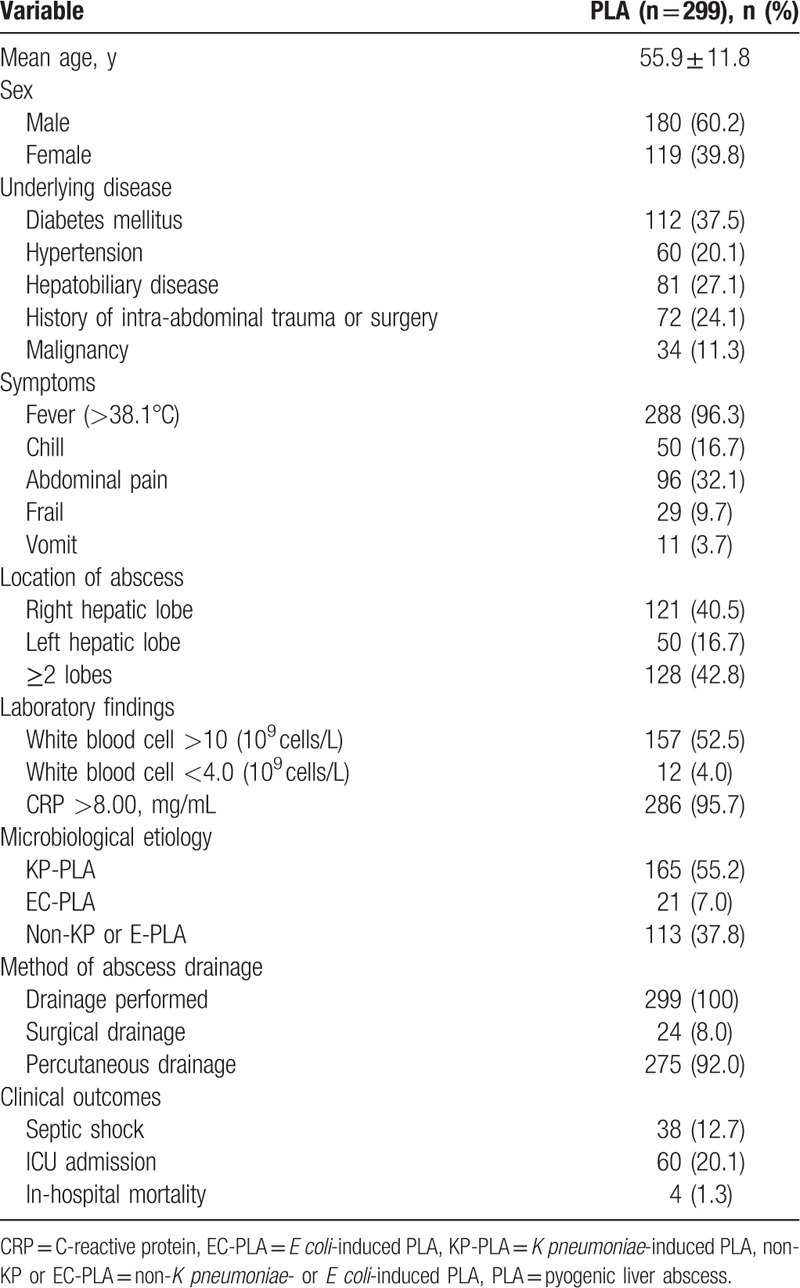
Demographic characteristics and clinical features of 299 patients with PLA at a tertiary teaching hospital.

### 3.2 Bacteriology

A total of 217 (72.6%) patients with PLA had an identifiable organism and 228 strains were identified from blood or pus culture. These included 24 Gram-positive organisms, 198 Gram-negative organisms, and 6 anaerobes (Table 2). Among culture-positive patients, 206 (94.9%) had monomicrobial infection, whereas 11 (5.1%) had polymicrobial infection. Among all isolates, *K pneumoniae* was the most commonly isolated pathogenic bacterium, found in 76.0% (n = 165) of the culture-positive PLA patients, followed by *E coli* (n = 21, 9.7%), *Enterococcus* (n = 9, 4.1%), *Streptococcus* (n = 8, 3.7%), and *Staphylococcus* (n = 6, 2.8%). Polymicrobial infections here included *K pneumonia* plus *E coli*, *K pneumonia* plus *Enterobacter cloacae*, *K pneumonia* plus *Pseudomonas aeruginosa*, *E coli* plus *P aeruginosa*, *E. coli* plus *Enterococcus faecium*, *E coli* plus *Enterobacter aerogenes*, *E coli* plus *Edwardsiella tarda*, *Proteus mirabilis* plus *E faecium*, *Acinetobacter baumanmii* plus *Staphylococcal aureus*, *Citrobacter koseri* plus S*erratia marcescens*, and *P aeruginosa* plus *E faecium.*

**Table 2:**
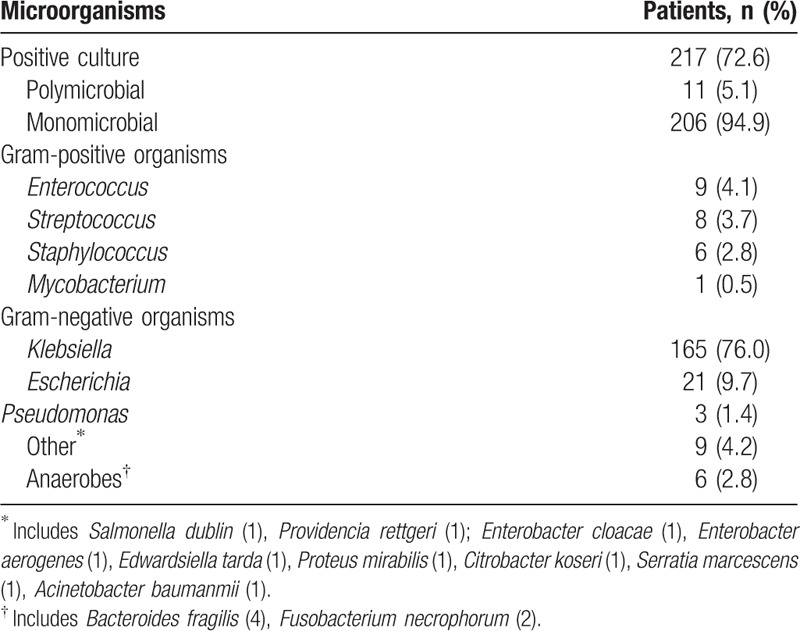
Microbiology of cultures (blood or pus) from patients with pyogenic liver abscesses in the hospitals.

### 3.3 Comparison of patients with KP-PLA and non-KP-PLA

Considering that *K pneumonia* has been found to be the primary pathogen in PLA, clinical features of KP-PLA and non-KP-PLA in 217 cases with positive culture results were further explored (Table 3). There was no significant difference in age or sex among patients. As for treatment strategies, laboratory findings, symptoms, number of abscesses, and location of abscess indicated no obvious difference between the 2 groups (*P* > .05). From medical chart review of KP-PLA patients, it was notable that compared with non-KP-PLA patients, there was a significant higher incidence of underlying metabolic disorders, such as diabetes mellitus (43.0% vs 21.2%, *P* = .005). To the contrary, it was less prevalent for concomitant hepatobiliary disease (20.0% vs 34.6%, *P* = .039) as well as history of intra-abdominal trauma or surgery (13.3% vs 38.5%, *P* < .001) in KP-PLA patients. Then we compared the clinical outcomes of KP-PLA and non-KP-PLA and found that more KP-PLA patients developed septic shock (10.9% vs 3.8%), admitted to ICU (22.4% vs 15.4%), or died in-hospital (2.4% vs 0%).However, these findings were not statistically significant.

**Table 3:**
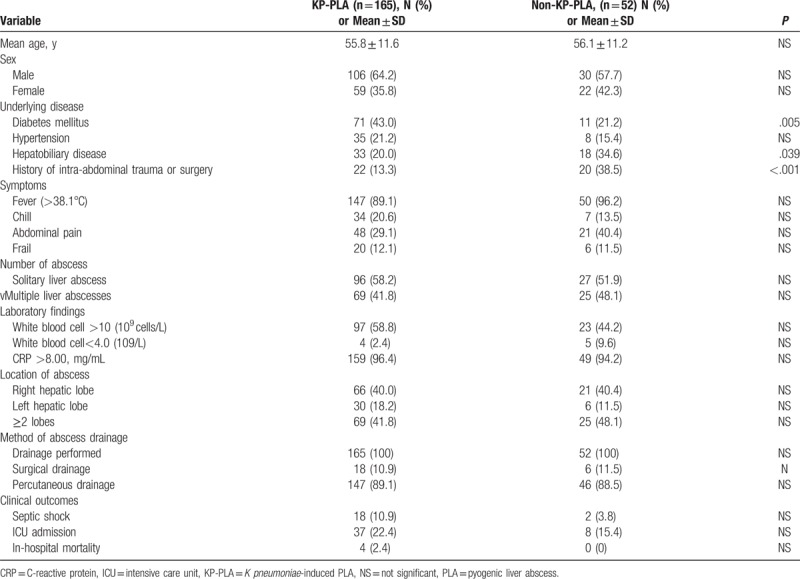
Comparison of demographic characteristics and clinical features of 217 culture-positive patients with KP-PLA versus non-KP-PLA.

### 3.4 Bacterial drug resistance

**Figure 1:**
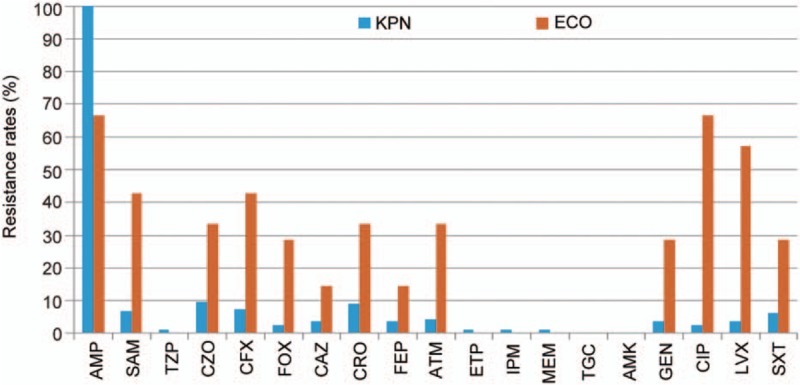
Antibiotic resistance rates of the *Klebsiella pneumonia* and *Escherichia coli* strains isolated from PLA patients. AMP = ampicillin, ATM = aztreonam, AMK = amikacin, CAZ = ceftazidime, CFX = cefuroxime, CIP = ciprofloxacin, CRO = ceftriaxone, CZO = cefazolin, ECO = *Escherichia coli*, ETP = ertapenem, FEP = cefepime, FOX = cefoxitin, GEN = gentamicin, IPM = imipenem, KPN = *Klebsiella pneumonia*, LVX = levofloxacin, MEM = meropenem, SAM = ampicillin/sulbactam, SXT = sulfamethoxazole/trimethoprim, TGC = tigecycline, TZP = piperacillin/tazobactam .

Given that *K pneumonia* and *E coli* were found to be major causative pathogens in PLA, 186 bacterial pathogens (165 *K pneumoniae* and 21 *E coli*), which were cultured and isolated from PLA specimens, were analyzed. The isolated *K pneumoniae* strains were susceptible to most of the common antibiotics that were used at the clinic, with low rates of resistance (<10%) except for ampicillin (Fig. 1). Only 11 (6.7%) strains of *K pneumoniae* were extended-spectrum β-lactamases (ESBLs) production and 2 (1.2%) strains developed resistance to carbapenems such as imipenem, ertapenem, and meropenem. Unlike *K pneumoniae*, *E coli*, which is the second most common isolate, has higher rates of resistance to most antibiotics other than ampicillin and higher ESBLs production (42.9%) than that of *K pneumonia* (6.8%). None of the isolates were found to be resistant to amikacin or tigecycline in all isolated *K pneumonia* and *E coli* strains in this center.

## 4 Discussion

The 3 major forms of hepatic abscess, classified by etiology, are pyogenic, amoebic, and fungal. PLA is a potentially life-threatening disease in many parts of the world. Given the prevalence of PLA and its severe complications, there is a need for early detection and appropriate treatment strategy for this disease. Ultrasonography and other imaging examination are critical means to diagnose liver abscess, but microbiological diagnosis is absolutely essential to establishing a causal relationship and strategizing further therapeutic plans.^[9]^ The most common pathogens of the pyogenic hepatic abscesses are *E coli*, *K pneumoniae*, *Bacteroides*, *Enterococci*, *Streptococci*, and *Staphylococci*.^[1]^ Up to now, *K pneumoniae* has been believed to surpass *E coli* to become the predominant cause of PLA during the past 3 decades. Our data confirmed that *K pneumoniae* is the predominant pathogen associated with PLA and was found in 76.0% (n = 165) of the culture-positive PLA patients, followed by *E coli* (n = 21, 9.7%).

*K pneumoniae* is an important cause of community and nosocomial-acquired infection worldwide. KP-PLA has been reported with increasing frequency in East Asian countries. PLA and KP-PLA are also health problems in mainland China^[10]^ and are in accordance with latest epidemiological trends observed in other countries and regions.^[11–14]^ We investigated characteristics in patients with PLA and KP-PLA in a tertiary teaching hospital during the period of 5 years. Our study manifested that KP-PLA was closely related to diabetes mellitus. Although diabetes mellitus is a known clinical inducing factor of KP-PLA, areal variation and demographic shifts contribute to different incidences of diabetes mellitus in KP-PLA patients. In the present study, 43.0% of KP-PLA patients were diabetic, which indicated that diabetes mellitus is associated with the development of PLAs. Clinicians should be highly alert of its clinical characteristics to optimize patient management.

Gastrointestinal colonization appears before KP-PLA. Overgrowth of *K pneumoniae* in the intestine that predisposes the patient to KP-PLA may be caused by use of amoxicillin/ampicillin. It was confirmed in clinic that to start ampicillin/amoxicillin therapy within the latest 30 days was related to an increase in KP-PLA risk.^[15]^ It was also suggested in animal study that oral ampicillin, disrupting the intestinal microflora but short of activity against *K pneumonia*, may result in a promotion of KP-PLA in *K pneumonia*-colonized mice.^[15]^ Given this, overuse of these antibiotics should be avoided in general practice, and besides, antibiotic administration should be improved to prevent undesired KP-PLA in endemic areas.

PLA caused by a gas-forming organism, such as *K pneumoniae*, usually follows a serious fulminant course and its associated morbidity and mortality are unacceptably likely unless immediate therapeutic interventions are initiated. Gas accumulation impairs the transportation of gases and nutrients in the local tissues and promotes tissue destruction to the point of abscess.^[16]^ Liver abscess caused by gas-forming organisms carry a high mortality and warrants immediate therapeutic intervention, which may include decisive surgical management and dedicated ICU. In our data, more patients with KP-PLA developed septic shock (10.9% vs 3.8%) required ICU admission (22.4% vs 15.4%) or died in-hospital (2.4% vs 0%) than that of non-KP-PLA. This shows that prompt recognition of the condition and appropriate treatment is crucial for proper management.

In the present study, we found bacterial culture to be a sensitive way to look for pathogens (72.6% were culture-positive). It is preferable to obtain pus or blood samples from either fine needle aspiration or abscess drainage for bacteria identification before the use of empirical antibiotics. One of the most common causes of liver abscess, *K pneumoniae*, is susceptible to most antimicrobial agents other than ampicillin (shown in blue in Fig. 1) despite the considerable antibiotic consumption in mainland China, which may lead to changes in gut microbiotas. The emergence of carbapenem-resistant *K pneumoniae* in some strains may lead to final treatment failure. This rising trend in resistance also corresponds to data collected elsewhere in the world, which shows an increasing prevalence of carbapenem-resistant Enterobacteriaceae infections. As known strains of *K pneumoniae* (e.g., serotype K1) are becoming increasingly virulent,^[17]^ it is prudent to ensure sensitivity-directed antibiotics therapy during PLA treatment to prevent further development of antibiotic resistance.

Several limitations to this study should be acknowledged. First, the study is a single-center, retrospective analysis that might give rise to selection bias in the aspects of patient population admitted to our hospital and recall bias related to medical history. Secondly, low positive rate of anaerobes may be attributed to inappropriate culture techniques for microorganism identification. However, we presented a clear profile of the antibiotic resistance of dominating pathogenic bacteria in PLA, and reminded physicians that antibiotic resistance is critical because of the emergence of carbapenem-resistant *K. pneumoniae* in some strains.

In conclusion, PLA is an infectious disease common in mainland China that requires hospitalization. *K pneumonia* is the leading pathogen of PLA, and KP-PLA patients have a higher incidence of diabetes mellitus and poorer clinical outcome. We presented a clear profile of the antibiotic resistance of pathogenic bacteria in PLA. This analysis indicated that the microbiological spectrum of PLA has evolved, and the predominant pathogens associated with PLA are *K pneumoniae* and *E coli*. To ensure a precise estimate of the epidemiology of the pathogens, further large-scale studies or even a population-based study is needed.

## Acknowledgments

The authors thank LetPub (www.letpub.com) for its linguistic assistance during the preparation of this manuscript.
